# Predicting Novel Binding Modes of Agonists to β Adrenergic Receptors Using All-Atom Molecular Dynamics Simulations

**DOI:** 10.1371/journal.pcbi.1001053

**Published:** 2011-01-06

**Authors:** Stefano Vanni, Marilisa Neri, Ivano Tavernelli, Ursula Rothlisberger

**Affiliations:** Laboratory of Computational Chemistry and Biochemistry, Ecole Polytechnique Fédérale de Lausanne, Lausanne, Switzerland; University of Houston, United States of America

## Abstract

Understanding the binding mode of agonists to adrenergic receptors is crucial to enabling improved rational design of new therapeutic agents. However, so far the high conformational flexibility of G protein-coupled receptors has been an obstacle to obtaining structural information on agonist binding at atomic resolution. In this study, we report microsecond classical molecular dynamics simulations of β_1_ and β_2_ adrenergic receptors bound to the full agonist isoprenaline and in their unliganded form. These simulations show a novel agonist binding mode that differs from the one found for antagonists in the crystal structures and from the docking poses reported by in silico docking studies performed on rigid receptors. Internal water molecules contribute to the stabilization of novel interactions between ligand and receptor, both at the interface of helices V and VI with the catechol group of isoprenaline as well as at the interface of helices III and VII with the ethanolamine moiety of the ligand. Despite the fact that the characteristic N-C-C-OH motif is identical in the co-crystallized ligands and in the full agonist isoprenaline, the interaction network between this group and the anchor site formed by Asp(3.32) and Asn(7.39) is substantially different between agonists and inverse agonists/antagonists due to two water molecules that enter the cavity and contribute to the stabilization of a novel network of interactions. These new binding poses, together with observed conformational changes in the extracellular loops, suggest possible determinants of receptor specificity.

## Author Summary

G-protein coupled receptors are the largest family of membrane proteins in the human genome and they constitute the largest class of drug targets. Amongst them, beta adrenergic receptors are involved in the regulation of muscular and vascular tone and are thus molecular targets for the treatment of various diseases including hypertension, heart failure and asthma. The function of these receptors is regulated via the binding of endogenous or exogenous ligands that can either lead to activation (agonists) or inactivation (inverse agonists/antagonists). However, structure determination of these receptors has been very elusive, and the few atomic resolution structures that are available so far have only been obtained in the presence of inverse agonists or antagonists. In order to study the binding mode of agonists inside the binding pocket, we employ all-atom molecular dynamics. This facilitates the study of the details of the interaction between agonist and receptor in full atomistic detail. We find that agonists binding to beta adrenergic receptors require the formation of a highly structured hydrogen bond network that is further stabilized by the presence of internal water molecules. The observed local rearrangements also help provide insights into the molecular origin of the differences between agonist and inverse agonist binding.

## Introduction

Beta adrenergic receptors are a class of transmembrane receptors responsible for binding catecholamines, such as the endogenous hormone adrenaline or the neurotransmitter noradrenaline. They belong to the G-protein-coupled receptors (GPCRs) family and are crucially involved in heart muscle contraction (β_1_), smooth muscle relaxation (β_2_) and lipolysis enhancement (β_3_). As a consequence, their signaling pathways are central for cardiac function regulation and relaxation of vascular and bronchial tone. The development of a large number of compounds able to modulate the activity of such receptors has been a major goal for the pharmaceutical industry to improve the clinical treatment of various diseases including hypertension, heart failure, asthma and preterm labor [1].

Since distinction between β adrenergic receptors can be based upon their relative affinities for the endogenous catecholamine agonists adrenaline and noradrenaline, determination of the differences that are responsible for their characteristic role upon agonist activation is crucial for the development of selective β-blockers [2].

The pharmacological characteristics of adrenergic receptors and their relative affinities and efficacies have been studied exhaustively, leading to the identification of a large number of clinically relevant agonists and antagonists. However, only recently determination of the crystal structures of β_2_ and β_1_ adrenergic receptors bound to inverse agonists/antagonists has provided a view of the binding mode of ligands inside the orthosteric binding pocket with atomic resolution [3], [4]. In particular, these crystal structures have confirmed that the crystallized ligands are engaged in specific interactions with a set of amino acid side chains in helices III, V, VI and VII that extensive mutation analyses already suggested as preferred interaction partners for catecholamines [5], [6], [7], [8], [9], [10]. In addition, the X-ray data suggested a functional role for the second extracellular loop (ECL2), based on its structure and close proximity with the bound ligand.

An atomistic description of the binding mode of agonists, on the other hand, is still lacking, and structure determination of adrenergic receptors in complex with agonists has so far been proven elusive. To address this pharmacologically crucial issue, structure-based drug design using the antagonist-bound β_2_AR structure as a template have been recently reported [11], [12], [13], [14], [15], [16]. These studies have primarily focused on the ability to identify partial/full agonists with docking based in silico screening methods, focusing on the molecular description of the strong agonist-specific [6], [10] polar interaction network between the catechol functional group and an anchor site formed by three serines in helix V, and the possible displacement of this helix to ease agonist binding [13], [16].

However, it is widely acknowledged [10], [17], [18], [19] that agonist binding is an intrinsically dynamical event that occurs via kinetically distinguishable conformational intermediates [20], and indeed recent in silico screening of approximately 1 million of commercially available “lead-like” molecules has confirmed an apparent bias toward inverse agonists among the docking hits [14].

On the other hand, it is known that agonist efficacy can be modulated by a number of allosteric factors, including G protein binding [21], GDP and GTP concentration [21], pH [22] and oligomerization state [23]. In particular, recent NMR studies on rhodopsin [24] and on β_2_AR [25] have revealed that the conformation of the extracellular surface of these receptors changes upon activation and that, in β_2_AR, drugs exhibiting different efficacies towards G-protein activation can stabilize distinct conformations of the extracellular loops, thus demonstrating a conformational coupling between this region and the orthosteric binding site. These findings are of special interest in view of the fact that the binding sites are very similar amongst β adrenergic receptors, whereas the extracellular loops are remarkably diverse and are therefore a possible target for the discovery of subtype-selective drugs.

To further elucidate agonist binding in the family of β adrenergic receptors, taking into account inherent receptor flexibility and explicit solvation known to be crucial for GPCR function [26], [27], [28], [29], we have carried out submicrosecond MD simulations of β_1_ and β_2_ adrenergic receptors bound to the potent agonist isoprenaline as well as in their apoforms. In order to properly analyze the agonist-induced local conformational changes in the two receptors, we also compare these simulations with previously reported MD simulations of β_1_ and β_2_ adrenergic receptors bound to the antagonist cyanopindol and the to the inverse agonist carazolol [30].

Anticipating our results, our simulations suggest that internal water molecules, that are usually left out in rigid docking experiments, play a major role in stabilizing agonist-receptor interactions, participating in two complex hydrogen bond networks between the agonist and the receptor. One of them involves the catechol moiety of the agonist while the other its ethanolamine part, and both differ from the inverse agonist interactions reported in the recently solved crystal structures of β adrenergic receptors [3], [4]. In addition, the specific behavior of the extracellular loops helps rationalize the allosteric activity of this region and provides meaningful insights into drug-receptor specificity.

## Methods

All simulations are based on the crystal structure of human β_2_ Adrenergic Receptor (Protein Data Bank code: 2RH1) [3], and on chain B of the crystal structure of partially mutated (β_1_AR-m23) turkey β_1_ Adrenergic Receptor (Protein Data Bank code: 2VT4) [4]. Missing amino acids (including the third extracellular loop and the C and N termini) and ionizable side chains have been modeled according to Ref. [30]. In β_1_AR, residues S68, V90, A227, L282, A327, M338 are mutated back to R68, M90, Y227, A282, F327 and F338.

The explicit membrane environment is formed by 1-stearoyl-2-oleoyl-*sn*-glycero-3-phosphoethanolamine (SOPE) lipids, and the systems are immersed in a box of SPC water [31]. Sodium and chloride ions were added to the aqueous phase to obtain an overall neutral system at physiological ion concentration. The systems consist of approximately 100.000 atoms in a box of size 100 cubic Å.

**Figure 1:**
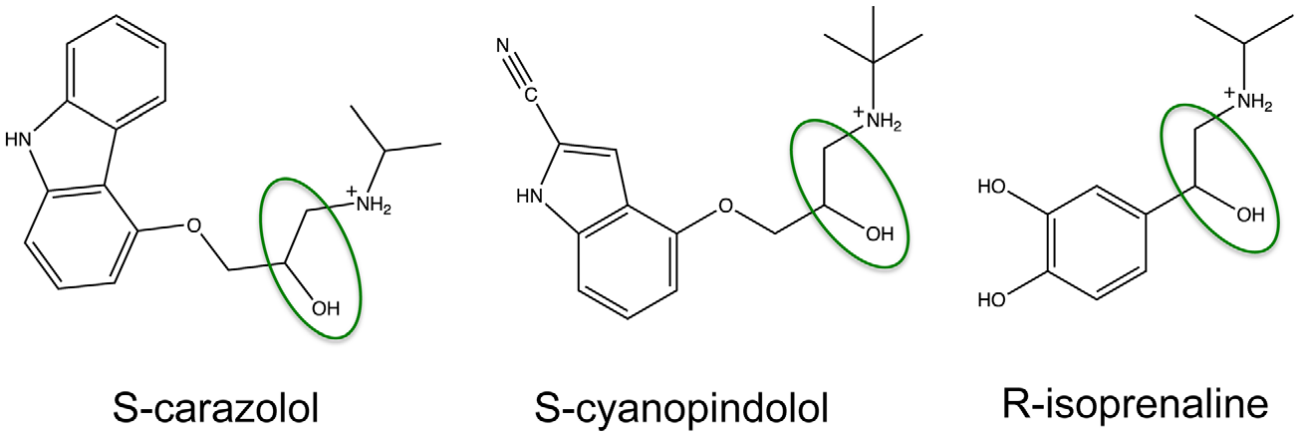
Chemical structures of adrenergic ligands. Chemical structures of the co-crystallized inverse agonists S-carazolol (left) and S-cyanopindolol (center) and of the full agonist R-isoprenaline (right). In the green oval, the C-C-O-H motif discussed in the Results section is highlighted.

The all-atom AMBER/parm99SB [32] force field was used and all bound ligands (S-carazolol, S-cyanopindolol and R-isoprenaline) carry a net positive charge of +1e (see Figure 1). The atomic charges for these ligands were derived by RESP [32], [33], [34] fitting using HF/6-31G* optimized structures and electrostatic potentials obtained using the Gaussian03 package [35]. The forcefield parameters for the ligands are reported in Supplementary Information (Dataset S1, S2 and S3).

All data collections and equilibration runs were done using GROMACS 4 [36]. Electrostatic interactions were calculated with the Ewald particle mesh method [37], with a real space cutoff of 12 Å. Bonds involving hydrogen atoms were constrained using the LINCS [38] algorithm and the time integration step was set to 2 fs. The systems were coupled to a Nosé-Hoover thermostat [39], [40] and to an isotropic Parrinello-Rahman barostat [41] at a temperature of 310K and a pressure of 1 atm.

Simulations of the apoforms and isoprenaline-bound β_2_AR and β_1_AR were started from the equilibrated carazolol-bound and cyanopindolol-bound structures taken from Ref. [30] after removal of the bound ligand or replacement of the bound carazolol with isoprenaline using a superposition of the N-C-C-OH motif shared by many adrenergic receptor agonists and antagonists. The systems were then slowly heated up to 310 K in 1040 ps without restraints.

Data analysis was performed on the following systems (between parenthesis the length of the corresponding MD runs in the case of a deprotonated Asp(2.50) and of a protonated Asp(2.50)) for a cumulated length of 6.5 µs: carazolol-bound β_2_AR (820 ns; 600 ns), isoprenaline-bound β_2_AR (830 ns; 500 ns), unliganded β_2_AR (800 ns; 450 ns); cyanopindolol-bound β_1_AR (820 ns; 600 ns), isoprenaline-bound β_1_AR (500 ns; 500 ns), unliganded β_1_AR (500 ns). Unless stated otherwise, the analyses described in the text refer to the simulations with deprotonated Asp(2.50).

All data analysis were done using GROMACS [36] utilities and all molecular images were made with Visual Molecular Dynamics (VMD) [42]. Hydrogen bonds are defined by a heavy atom distance cutoff of 3 Å and an angle cutoff of 20 degrees.

## Results

### Orthosteric binding site

Comparison of the chemical similarities of β adrenergic receptor ligands suggests that while some interactions might be common for agonists and antagonists, others can be expected to be specific for agonists only. In particular, while most β adrenergic agonists and antagonists (including the co-crystallized cyanopindolol and carazolol) present a positively charged amine or ethanolamine groups, the presence of the polar catechol group is strongly agonist specific.

At the same time, it is known that while antagonist binding to β adrenergic receptors is largely entropy driven, with only a small enthalpy component, the binding of agonists is associated with a large decrease in enthalpy [43]. These considerations suggest formation of a large structured hydrogen bond network, probably located in close proximity to Ser(5.42) [10], Ser(5.43) [6] and Ser(5.47) [6] in helix V, as possible key component of agonist binding.

The crystal structures of the antagonist/inverse agonist bound forms have indeed confirmed these considerations, showing that the carbazole heterocycle of carazolol and the indole moiety of cyanopindolol interact with the receptor mainly via hydrophobic interactions and a lone hydrogen bond with Ser(5.42), while Asp(3.32) and Asn(7.39) form a complementary H-bond network with the ethanolamine group of the ligands.

To understand the binding mode of agonists inside the binding pocket of adrenergic receptors as well as the conformational changes induced in the receptor by the presence of different ligand effectors, we performed MD simulations ranging between 500 ns–830 ns of β_1_ and β_2_ adrenergic receptors in their apoforms, and bound to the full agonist isoprenaline. The simulations were started from receptor structures bound to the co-crystallized antagonist cyanopindolol and the inverse agonist carazolol previously equilibrated in an explicit membrane environment (see Methods and ref. [44]).

**Figure 2:**
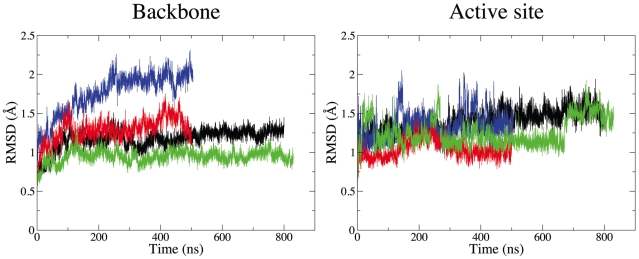
Transmembrane region and binding pocket root mean square deviation. Root mean square deviation (RMSD) of backbone atoms of all alpha helices (left) and of residues in the binding site (right) for unliganded β_2_AR (black), unliganded β_1_AR (blue), isoprenaline-bound β_2_AR (green) and isoprenaline-bound β_1_AR (red).

Root mean square deviation (RMSD) analysis of the backbone atoms of all alpha helices as well as of the ligand binding site, defined as all residues within 5 Å from the bound ligand in the crystal structures, suggests that the simulations are equilibrated within approximately one hundred nanoseconds (see Figure 2). Very little global structural rearrangements with respect to the crystal structures, in line with previously reported MD simulations of the same systems in their apo [45] and antagonists-bound [44] forms, are observed.

At the same time, the apoforms of the receptors need a longer equilibration time, especially in the case of β_1_AR, where equilibration is reached only after approximately 200 ns. This difference is related to the extent of internal solvation-induced rearrangements that take place in the apoform, in contrast to the case of the ligand bound receptors where a set of hydrophobic residues contributes to ligand stabilization inside the binding pocket with only few internal water molecules playing a crucial role.

Interestingly, the ligand binding site remains very close to the original conformation both in MD simulations of the isoprenaline-bound forms and in the unliganded systems, suggesting that only local rearrangements take place. The most significant of these local changes are due to conformational transitions of crucial residue Phe193 in the second extracellular loop (ECL2) which cause the fluctuations of the active site RMSD in the isoprenaline-bound β_2_AR simulations (green line in the right panel of Figure 2) and their functional significance will be discussed in more detail below.

#### Agonist interactions with helices V–VI

**Figure 3:**
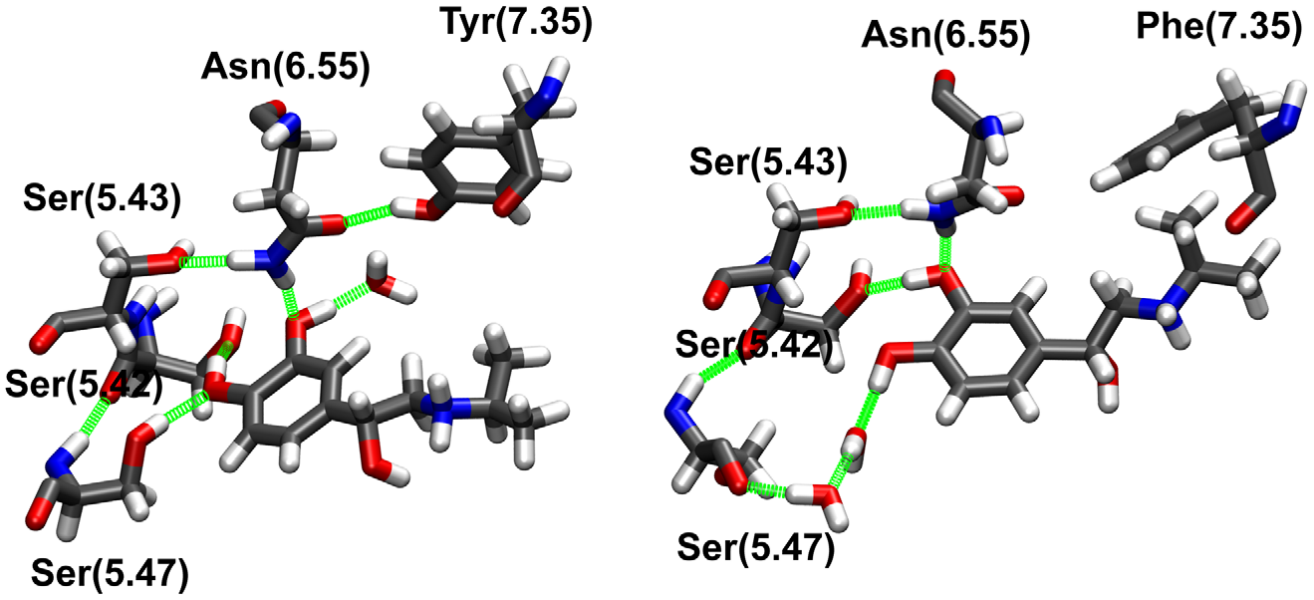
Agonist interactions with helices V and VI. MD snapshots of the hydrogen-bond interaction network between isoprenaline and Ser(5.42), Ser(5.43), Ser(5.47), Asn(6.55), Tyr/Phe(7.35) and internal water molecules in β_2_AR (left) and β_1_AR (right).

After equilibration, the catechol moiety of isoprenaline is engaged in a stable hydrogen bond network (see Figure 3 and Table S1 of Supplementary Information) formed by the two hydroxyl groups of the ligand with the side chains of Ser(5.42) [10], Ser(5.43) [6] and Ser(5.47) [6], as well as Asn(6.55) [8].

In particular, the Ser(5.42) side chain oxygen directly hydrogen bonds one of the two hydroxyl groups of isoprenaline, while Ser(5.47) can either form a direct H-bond with one hydroxyl group of the ligand (in β_2_AR) or a water mediated hydrogen bond network with the catechol moiety (β_1_AR). On the other hand, Ser(5.43) is not directly interacting with the bound ligand but instead stabilizes the side chain conformation of Asn(6.55) via an hydrogen bond between its side chain oxygen and one of the two hydrogen atoms of the NH_2_ moiety of Asn(6.55), restraining the conformation of the other hydrogen atom of Asn(6.55) side chain to form an additional H-bond with a catechol oxygen of isoprenaline. This conformation of Asn(6.55) is further stabilized in β_2_AR by an additional hydrogen bond between its side chain oxygen and the hydroxyl group of Tyr(7.35) that is mutated into a phenylalanine residue in β_1_AR, the lone sequence difference between the two receptors in the orthosteric binding site. Interestingly, despite the different behaviour of Asn(6.55) in the two systems (as suggested by the time evolution of the χ_2_ angle along the dynamics, see Figure S1 of Supplementary Information) the most stable conformation is the same for both the β_1_ and the β_2_.

The two systems differ, however, in the degree of hydration of the catechol-helix V interaction network: while in β_2_AR waters are sequestrated and can interact with the catechol moiety only in the extracellular side of the binding pocket, in β_1_AR two water molecules play a crucial role in modulating the interaction between the drug and the serine triplet, mainly via Ser(5.47). These two internal water molecules are present during the entire simulation time in both systems (β_1_AR and β_2_AR) at the interface between helices III and V but do interact directly with isoprenaline only in the β_1_AR simulation.

Interestingly, a recent in silico docking study on β_2_AR, has reported that motion of helix V relatively to the binding pocket could produce a marked enhancement of the calculated binding affinities for agonist compounds [13]. In the MD simulations of both systems, however, helix V shows very limited movement, suggesting that side chain reorientation is sufficient to achieve stable binding between receptor and agonist. It is likely that the absence of internal water molecules in the docking protocol could be responsible for unfavorable interactions (and thus scoring hits) with helix V in the crystal structure conformation, while, on the other hand, internal water molecules in the MD simulations play a significant role in the stabilization of the interaction network between agonist and receptor.

On the other hand, the relative distance between helix III and helix VI (described by the C_α_-C_α_ distance between Asp(3.32) and Asn(6.55)) decreases in both β_1_AR and β_2_AR by approximately 1 Å with respect to the crystal structures and MD simulations with inverse agonists. Even though the two residues remain far apart (the average distance between Asp(3.32) and Asn(6.55) is around 14.6±0.4 Å in the MD simulations), this movement seems to be in line with biophysical studies [8], [46] suggesting that the distance between these two residues should decrease during activation.

Interestingly, in the simulations of unliganded β_1_AR and β_2_AR the behavior of the relative distances between helix III and helices V and VI is remarkably different. While in β_2_AR both helix V and helix VI move away from helix III as a consequence of water entering the binding pocket, in β_1_AR the distance between helix III and helix VI slightly increases, while the distance between helix III and helix V decreases despite hydration of the cavity (Figure S2, Supplementary Information).

The conformational changes of the binding pocket in the two receptors are thus similar in the presence of isoprenaline, but different for the unliganded receptors. Isoprenaline is a potent full agonist for both β_1_ and β_2_ adrenergic receptors, while the two receptors show a considerably different amount of constitutive activity. Therefore, the different behavior of the binding pocket in the simulations could be linked to the specific activity of the two receptors under the effect of isoprenaline or in the absence of any external effector molecule.

#### Agonist interactions with helices II, III and VII

**Figure 4:**
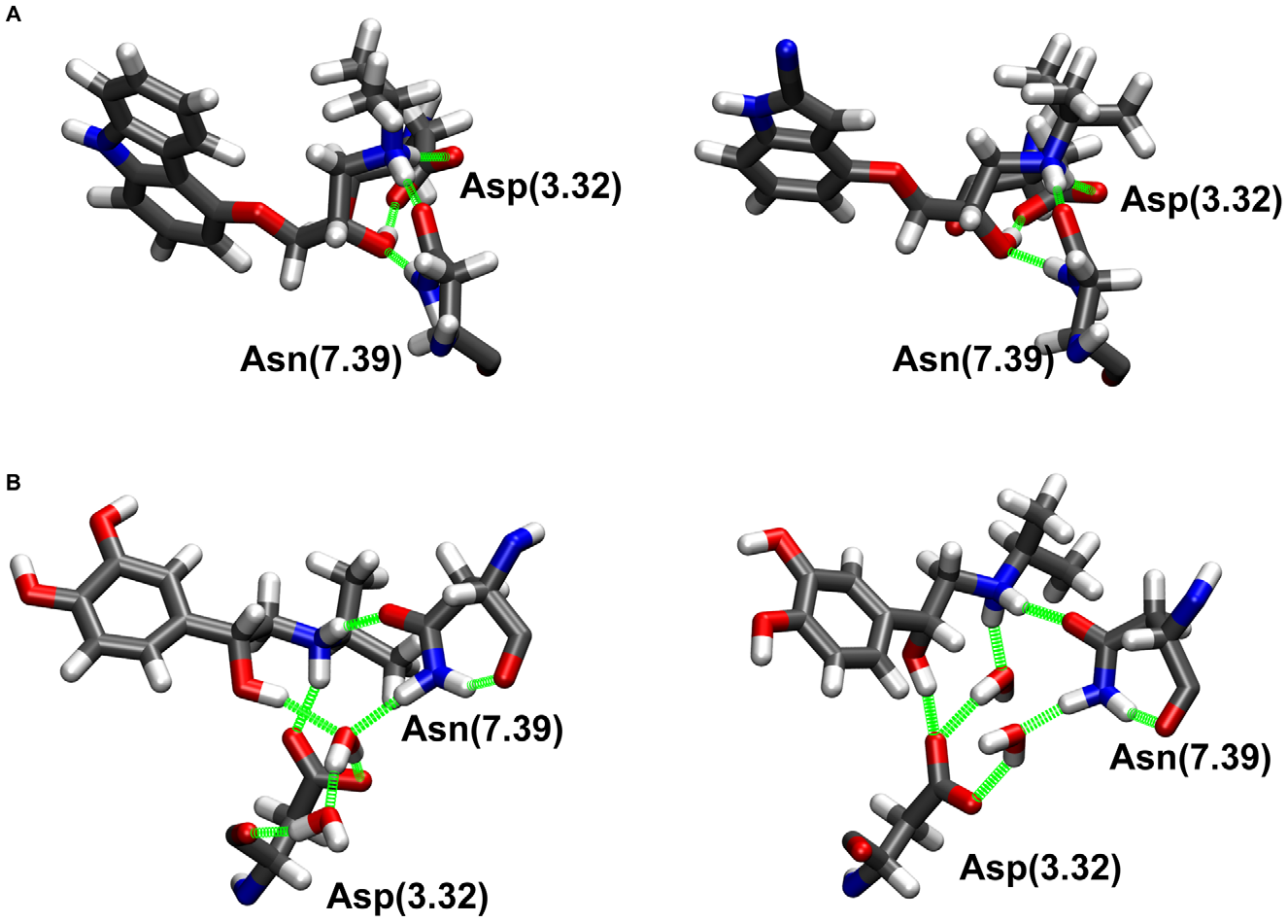
Agonist interactions with helices III and VII. Panel A: Hydrogen-bond interaction network between Asp(3.32) and Asn(7.39) and carazolol in β_2_AR (left) and cyanopindolol in β_1_AR (right). Panel B: MD snapshot of the hydrogen-bond interaction network between isoprenaline and Asp(3.32), Asn(7.39) and internal water molecules in β_2_AR (left) and β_1_AR (right).

On the opposite side, most adrenergic ligands (agonists, antagonists and inverse agonists) present a positively charged amine or ethanolamine group. The crystal structures of the co-crystallized inverse agonists suggest that this group forms a complementary H-bond network with Asp(3.32) and Asn(7.39) at the interface between helices II, III and VII, where Asn(7.39) acts both as a H-bond acceptor and donor to the amine nitrogen and hydroxyl oxygen of the ligands and Asp(3.32) is a H-bond acceptor for the protonated amine nitrogen and the hydroxyl group of the ligands (see Figure 4, panel A). This network remains stable during submicrosecond MD simulations of carazolol-bound β_2_AR and of cyanopindolol-bound β_1_AR [44].

Despite the fact that the characteristic N-C-C-OH motif is identical in the co-crystallized ligands and in the full agonist isoprenaline (see Figure 1), MD simulations show that the dynamical behavior of the network of interactions of this group is substantially different between agonists and inverse agonists/antagonist. In fact, two water molecules enter the cavity and contribute to the stabilization of a novel interaction network between the drug, Asp(3.32) and Asn(7.39) (Figure 4, panel B). These two water molecules are present in the cavity for almost 100% of the simulation time and they exchange with the bulk with a frequency of 120±60 ns^−1^. Furthermore, while the N-C-C-O dihedral angle remains in the g(−) conformation in all systems, the value of the C-C-O-H dihedral angle varies substantially amongst the different simulations (Table 1).

**Table 1:** Ligand C-C-O-H dihedral angle populations.

System	Dihedral conformation	Isoprenaline Asp(2.50) deprotonated	Isoprenaline Asp(2.50) protonated	Carazolol Asp(2.50) deprotonated	Carazolol Asp(2.50) protonated
β_2_AR	g(−)	26%	85%	84%	95%
β_2_AR	g(+)	70%	15%	16%	5%
β_2_AR	trans	4%	0%	0%	0%
β_1_AR	g(−)	42%	46%	46%	42%
β_1_AR	g(+)	0%	47%	54%	58%
β_1_AR	trans	58%	7%	0%	0%

Populations of the C-C-O-H dihedral angle of the ligand inside the binding pocket of β_2_AR and of β_1_AR along the MD simulations. The values of the dihedral angle are calculated within ±30° of the standard value (60° for g(−), −60° for g(+) and 180 for trans).

It has been recently proposed [30] that the protonation state of Asp(2.50), a conserved aspartic acid in the transmembrane core of the receptors, could be involved in receptor activation, suggesting that when Asp(2.50) is deprotonated the equilibrium between the active and the inactive state is shifted towards the active configuration while when the residue is protonated the equilibrium is shifted towards the inactive state. At the same time, mutagenesis experiments in β_1_ and β_2_ adrenergic receptors have also shown that mutations at position 2.50 not only affect receptor downstream signaling, but can also alter agonist affinity leaving antagonist affinity to the receptors unaltered [5], [47], [48], [49].

In Table 1, the values of the C-C-O-H dihedral angle (highlighted in Figure 1) of the bound ligand for both protonation states of Asp(2.50) are reported. The dihedral angle is strongly restrained in the g(−) conformation for the potent inverse agonist carazolol bound to β_2_AR, while an equilibrium between the g(−) and the g(+) conformation is present for cyanopindolol in β_1_AR. Interestingly, these two equilibria are not substantially altered upon changes of the protonation state of Asp(2.50), in agreement with the mutagenesis experiments showing that antagonist-binding is not substantially affected by mutations at position 2.50 [5], [47], [48], [49] in β_1_ and β_2_ adrenergic receptors.

On the other hand, the equilibrium conformation of the C-O-O-H dihedral angle is substantially altered upon changes in the protonation state of Asp(2.50) in the case of isoprenaline-bound β_1_AR and β_2_AR. When Asp(2.50) is protonated, the dihedral angle is maintained in a conformation similar to the one found in the simulations of inverse agonist bound receptors, while the equilibrium drastically shifts towards the g(+) conformation for the isoprenaline-bound β_2_AR and towards the trans conformation for the isoprenaline-bound β_1_AR if Asp(2.50) is deprotonated. Since the active state is indeed favored when Asp(2.50) is deprotonated [30], the simulations suggest that the network of interactions between isoprenaline and the helix III/helix VII interface in β adrenergic receptors can differ significantly from the one suggested by the crystal structure with inverse agonists.

In addition, simulations of unliganded β_1_AR and β_2_AR show spontaneous stable binding of a sodium ion to Asp(3.32) inside the binding pocket when Asp(2.50) is deprotonated. On the other hand, such an event never takes place when Asp(2.50) is deprotonated.

### Extracellular loops

The crystal structures of β adrenergic receptors have revealed that the structure of the extracellular loops in these receptors able to bind diffusible ligands is remarkably different from rhodopsin where the N-terminus and ECL2 form a structured cap over the covalently bound retinal to prevent ligand hydrolysis. In order to allow ligand access to the binding pocket, ECL2 and ECL3 in adrenergic receptors are mainly composed of polar and charged residues and, unlike in rhodopsin, they do not prevent ligand access, even though rearrangements of ECL2 are expected during ligand entry and exit [50]. Recent NMR studies on rhodopsin [24] and on β_2_AR [25] have revealed that the conformation of the extracellular surface changes upon activation and that, in β_2_AR, drugs exhibiting different efficacies towards G-protein activation can stabilize distinct conformations of the extracellular loops. All these findings demonstrate a conformational coupling between this region and the orthosteric binding site. In particular, it has been suggested that the extracellular Lys305-Asp192 salt bridge in β_2_AR (Figure S3, Supplementary Information) is weakened in the active state and that inverse agonists may function in part by stabilizing bulky hydrophobic interactions with Phe193 in ECL2 that block the motion of helix VI. These findings are of special interest because although the ECL2 and ECL3 backbone conformations are very similar in β_1_ and β_2_ adrenergic receptors, only 55% of their residues are identical, in contrast to the 94% sequence identity of the binding pockets.

**Figure 5:**
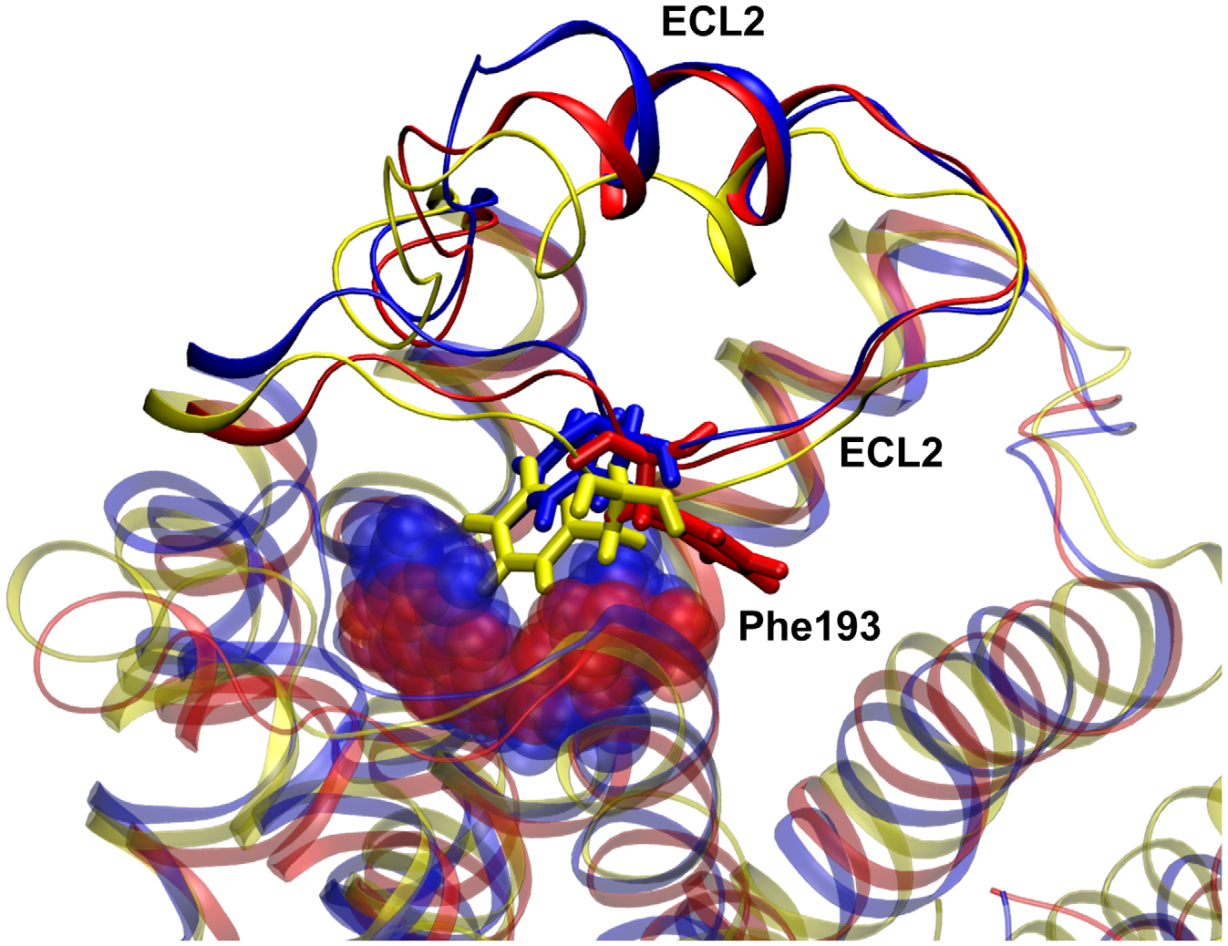
Conformations of second extracellular loop in β_2_AR. Conformation of the second extracellular loop (ECL2) and of Phe193 (sticks representation) in MD simulations of unliganded β_2_AR (yellow), carazolol-bound β_2_AR (blue) and isoprenaline-bound β_2_AR (red). Isoprenaline and carazolol are shown in transparency. Root mean square fluctuations are shown in Figure S5 of Supplementary Information.

Interestingly, while in the simulations of β_2_AR bound to carazolol and isoprenaline the backbone structure of ECL2 and its relative distance to TM7 remain approximately identical to the crystal structure, in the simulation of unliganded β_2_AR ECL2 approaches the binding pocket (see Figure 5). Notably, even if the salt bridge between Lys305 and Asp192 remains stable in all simulations (Figure S3, Supplementary Information), the conformation of Phe193 is substantially different in the three simulations (see Figure 5): it remains close to the crystal structure conformation in the carazolol-bound simulations (trans conformation), it partially displaces towards helix III and VII in apo-β_2_AR and it adopts g(+) and g(−) conformations interacting with the hydrophobic tail of the ligand in the isoprenaline-bound simulation (Figure S4, Supplementary Information). As a consequence of the displacement of Phe193 in the isoprenaline-bound case, the side chain of Thr195 changes orientation and its hydroxyl group points towards helix III eventually hydrogen bonding Phe193 backbone oxygen.

In β_1_AR, on the other hand, the salt bridge between Lys305 and Asp192 is absent, because lysine is replaced by the aspartic acid Asp322. However, the high degree of structural similarity of the backbone conformations of loops ECL2 and ECL3 in the two receptors suggests that the role of these charged residues (lysine and aspartic acid in β_2_AR and two aspartic acids in β_1_AR) is not directly related to loop stabilization. At the same time, the behavior of ECL2 is similar to the one observed in β_2_ receptor: ECL2 remains close to the crystal structure conformation in the isoprenaline-bound simulation, while it approaches the binding pocket in unliganded β_1_AR. At the same time, Phe201 in β_1_AR (which is equivalent to Phe193 in β_2_AR) is also approaching helix III in the isoprenaline-bound simulation but without changing side chain rotameric conformation.

**Figure 6:**
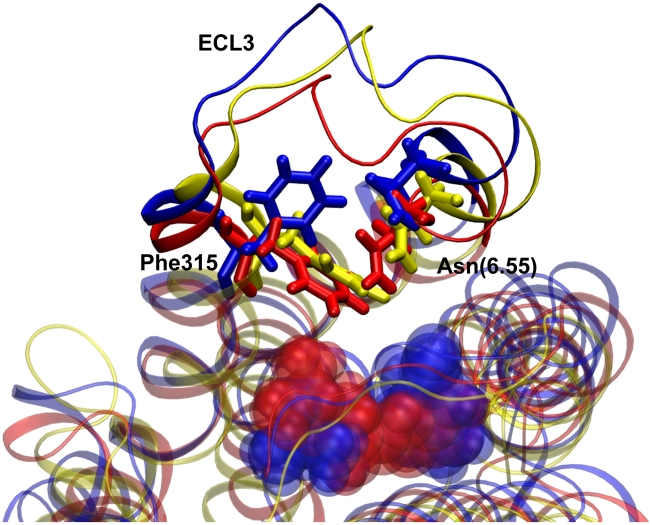
Conformations of third extracellular loop in β_1_AR. Conformation of the third extracellular loop (ECL3) and of Phe315 and Asn(6.55) (sticks representation) in MD simulations of unliganded β_1_AR (yellow), cyanopindolol-bound β_1_AR (blue) and isoprenaline-bound β_1_AR (red). Isoprenaline and cyanopindolol are shown in transparency.

Remarkably, while in the simulations of β_2_AR the overall structure of ECL3 remains very close to the crystal structure independently of the nature of the bound ligand, the behaviour of this loop is substantially different in the simulations of β_1_AR. In fact, in the cyanopindolol-bound simulation of β_1_AR, ECL3 is displaced from the binding site and Phe315 points towards the extracellular side moving away from the ligand interaction region. On the other hand, due to the additional interactions that are formed between the catechol moiety of isoprenaline and Asn(6.55), in the agonist-bound simulations ECL3 approaches the binding site, with Phe315 playing a prominent role in the hydrophobic stabilization of the binding site (see Figure 6). Since ECL3 is linking helices VI and VII, this event could be a precursor of an inward motion of the extracellular moiety of helix VI towards helix III to favor the interaction between Asn(6.55) and the β hydroxyl group of the agonist that is supposed to be a later intermediate along the activation pathway [8].

## Discussion

Even though a clear understanding of the binding mode of agonists to β adrenergic receptors would constitute a major step for the development of selective drugs, no structural information on agonist binding at atomic resolution is available yet and the only resolved crystal structures have been obtained in complex with inverse agonists or antagonists. As a consequence, the only available information on possible agonist docking poses can be inferred from rigid or semi-flexible docking protocols that use the inactive receptor as a template and suffer from well-known intrinsic limitations [12].

Even if the current capabilities of force-field based MD simulations do not allow to reach all intermediates along the activation pathway of adrenergic receptors, that are in the milliseconds time scale [46], they are able to follow the early local structural rearrangements that take place in the binding pocket due to the effect of agonist binding. Moreover, despite the limited statistic arising from the fact that only one replica per system was run, they allow determining the newly formed pattern of interactions between the bound ligand and the receptor taking correctly into account protein flexibility, allosteric modulation and internal solvation.

The microsecond MD simulations presented here show the formation of a complex hydrogen bond network between the catechol moiety of isoprenaline and a set of residues in helices V and VI, thus providing a possible explanation of the finding that agonist binding is associated with a large change in enthalpy, while antagonist binding is mainly entropy driven [43]. At the same time, they rationalize the role of Ser(5.43) [6] and Ser(5.47) [6] in agonist binding, while only Ser(5.42) [10] is involved in antagonist binding. Interestingly, despite being in close proximity to the bound ligand, Ser(5.43) does not interact directly with the drug, but stabilizes another crucial residue, Asn(6.55), through the formation of a stable hydrogen bond that restrains Asn(6.55) conformation enabling a direct interaction between the NH_2_ moiety of the residue and one of the two hydroxyls of the catechol group of isoprenaline. While it is acknowledged that Asn(6.55) is involved in agonist binding through the formation of an hydrogen bond with the β alcohol of the agonist in a late conformational stage, the simulations suggest that Asn(6.55) can also play a major role in agonist recognition in the early steps of the binding event.

In addition, the simulations do not support a large movement of helix V during agonist binding that was suggested based on the marked improvement in the calculated binding affinities for agonist compounds using a semi-flexible docking approach [13]. In contrast, it turns out that only very limited helix V movement is sufficient to achieve a very stable network of interactions that is a direct consequence of the presence of internal water molecules that help bridging the gap between the agonist and helix V.

In an analogous way, the presence of few internal water molecules plays a major role in the stabilization of the interaction between helices III and VII and the ethanolamine group of isoprenaline. Despite the structural and chemical similarity displayed by most agonists and antagonists, the binding mode of isoprenaline to Asp(3.32) and Asn(7.39) is remarkably different with respect to the antagonist binding mode suggested by the crystal structures, due to the presence of the internal water molecules. Interestingly, this decreased stability of the interaction between Asn(7.39) and the ethanolamine group of agonists was already reported by MD simulations of an endogenous agonist, adrenaline, where the newly formed interactions appeared to be dynamically less stable [51].

In addition, recent NMR studies on β_2_AR [25] have revealed a direct coupling between the extracellular loops and the ability of the receptor to activate its cognate G-protein, showing that different conformations of the extracellular loops can be stabilized upon binding of ligands with different activities. In particular, it has been suggested that the extracellular Lys305-Asp192 salt bridge in β_2_AR is weakened in the active state and that inverse agonists may function in part by stabilizing bulky hydrophobic interactions with Phe193 in ECL2 that block the motion of helix VI. Even though in our simulations we cannot observe any substantial change in the Lys305-Asp192 salt bridge, probably due to the fact that the time scales we are investigating are not sufficient to allow for a complete relaxation of the receptor to the active state, already in the submicrosecond time scale it is possible to notice a different behavior of Phe193 depending on the type of ligand that is bound to the receptor. The pronounced stability that Phe193 displays in the antagonist bound simulations (due to the presence of strong hydrophobic interactions) is lost in the unliganded and in the isoprenaline-bound simulations, and the conformational transitions of Phe193 side-chain allow for a closer interaction between this residue and the Lys305-Asp192 salt bridge, constituting a mean to potentially alter the strength of this salt bridge.

In conclusion, the reported microsecond MD simulations of agonist bound β adrenergic receptors propose a detailed and dynamical description of agonist-receptor interactions, where hydrogen bonding and internal water molecules play a crucial role. In addition, the specific behavior of the extracellular loops in the different systems can help rationalize the allosteric activity of such loops and provide possible clues into drug-receptor specificity.

## Supporting Information

Dataset S1 Isoprenaline topology file.
(0.01 MB TXT)

Dataset S2 Cyanopindolol topology file.
(0.02 MB TXT)

Dataset S3 Carazolol topology file.
(0.02 MB TXT)

Figure S1 Time evolution of Asn(6.55) χ2 angle in MD simulations of isoprenaline-bound β1AR (red line) and β2AR (green line).
(1.81 MB TIF)

Figure S2 Helix III-helix V (red) and helix III-helix VI (blue) distances in MD simulations of unliganded β1AR (left) and β2AR (right). The helix III-helix V distance is defined as the Cα-Cα distance between Asp(3.32) and Ser(5.43), while the helix III-helix VI distance is defined as the Cα-Cα distance between Asp(3.32) and Asn(6.55).
(0.76 MB TIF)

Figure S3 Left panel: Lys305-Asp192 salt bridge in β2AR. Time evolution of the Lys305-Asp192 salt bridge (Nζ@Lys305-Cγ@Asp192 distance) in MD simulations of carazolol-bound (blue), unliganded (yellow) and isoprenaline-bound (red) β2AR and respective frequency distribution. (red) β2AR.
(1.33 MB TIF)

Figure S4 Time evolution of the Phe193 χ1 angle in MD simulations of carazolol-bound (blue), unliganded (yellow) and isoprenaline-bound (red) β2AR.
(0.64 MB TIF)

Figure S5 Root mean square fluctuations of intracellular loop 2 in MD simulations of antagonist-bound (blue), unliganded (yellow) and isoprenaline-bound (red) β2AR (left) and β1AR (right). Atom index #1 corresponds to His172 in β2AR and to His180 in β1AR.
(0.49 MB TIF)

Table S1 Hydrogen bond network between isoprenaline and adrenergic receptors. The table shows the percentage of N-O or O-O distances below 3.2 angstrom of the hydrogen bonds shown in Figures 3 and 4 after equilibration.
(0.04 MB DOC)
