# Supplementary figures and images for: Predicting Novel Binding Modes of Agonists to β Adrenergic Receptors Using All-Atom Molecular Dynamics Simulations
Source: PLoS Comput Biol. 2011 Jan 6;7(1):e1001053. doi: 10.1371/journal.pcbi.1001053 (PMC3017103; doi:10.1371/journal.pcbi.1001053)

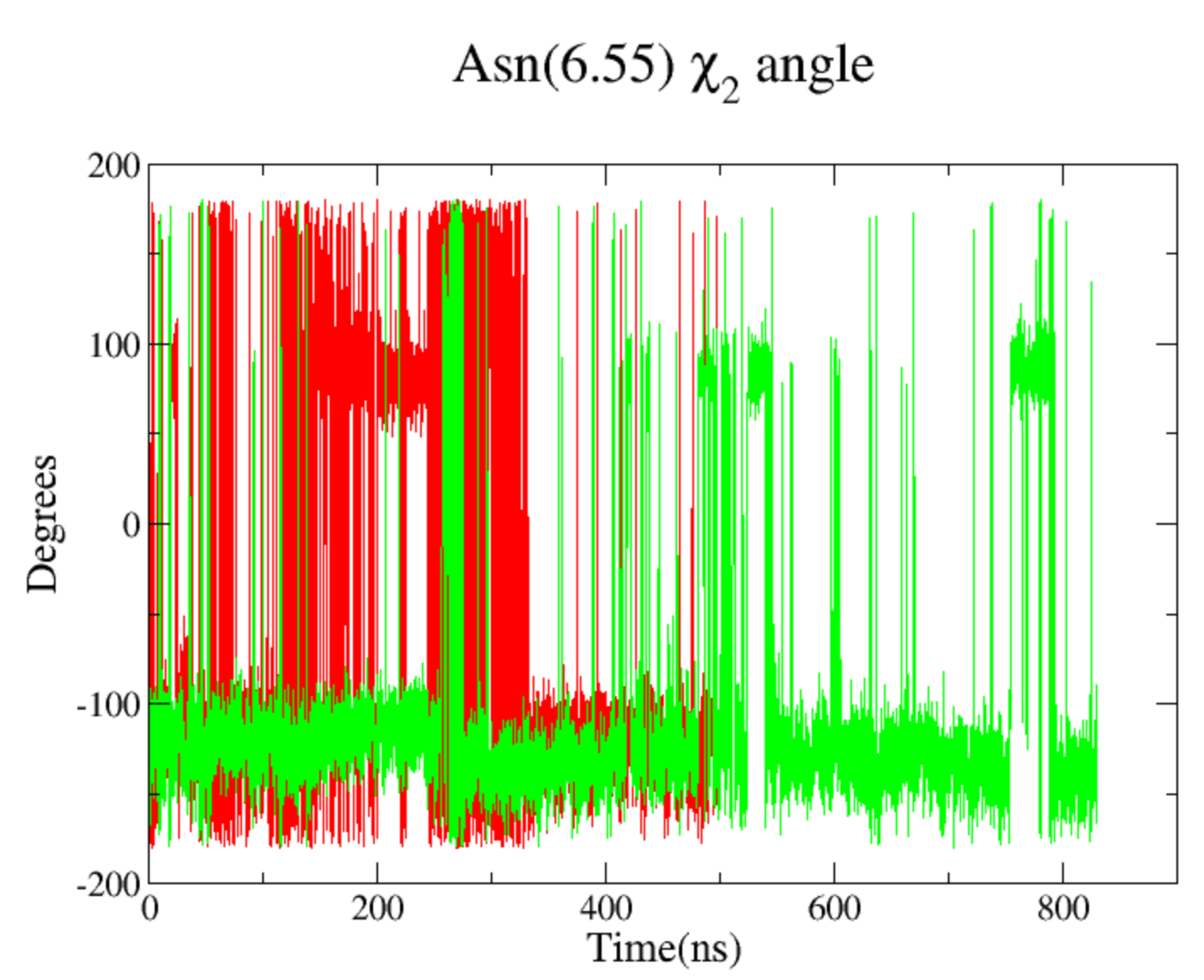

Supplement: Figure S1 — Time evolution of Asn(6.55) χ2 angle in MD simulations of isoprenaline-bound β1AR (red line) and β2AR (green line). (1.81 MB TIF) [file pcbi.1001053.s004.tif]

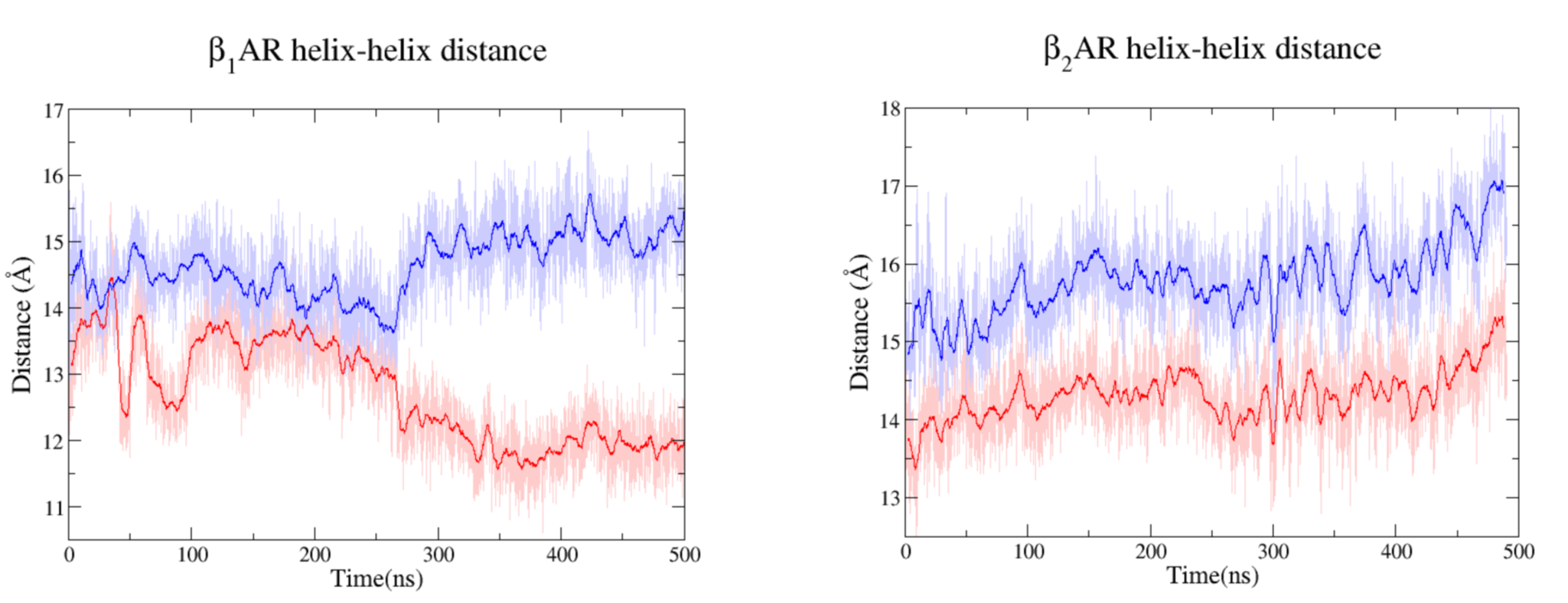

Supplement: Figure S2 — Helix III-helix V (red) and helix III-helix VI (blue) distances in MD simulations of unliganded β1AR (left) and β2AR (right). The helix III-helix V distance is defined as the Cα-Cα distance between Asp(3.32) and Ser(5.43), while the helix III-helix VI distance is defined as the Cα-Cα distance between Asp(3.32) and Asn(6.55). (0.76 MB TIF) [file pcbi.1001053.s005.tif]

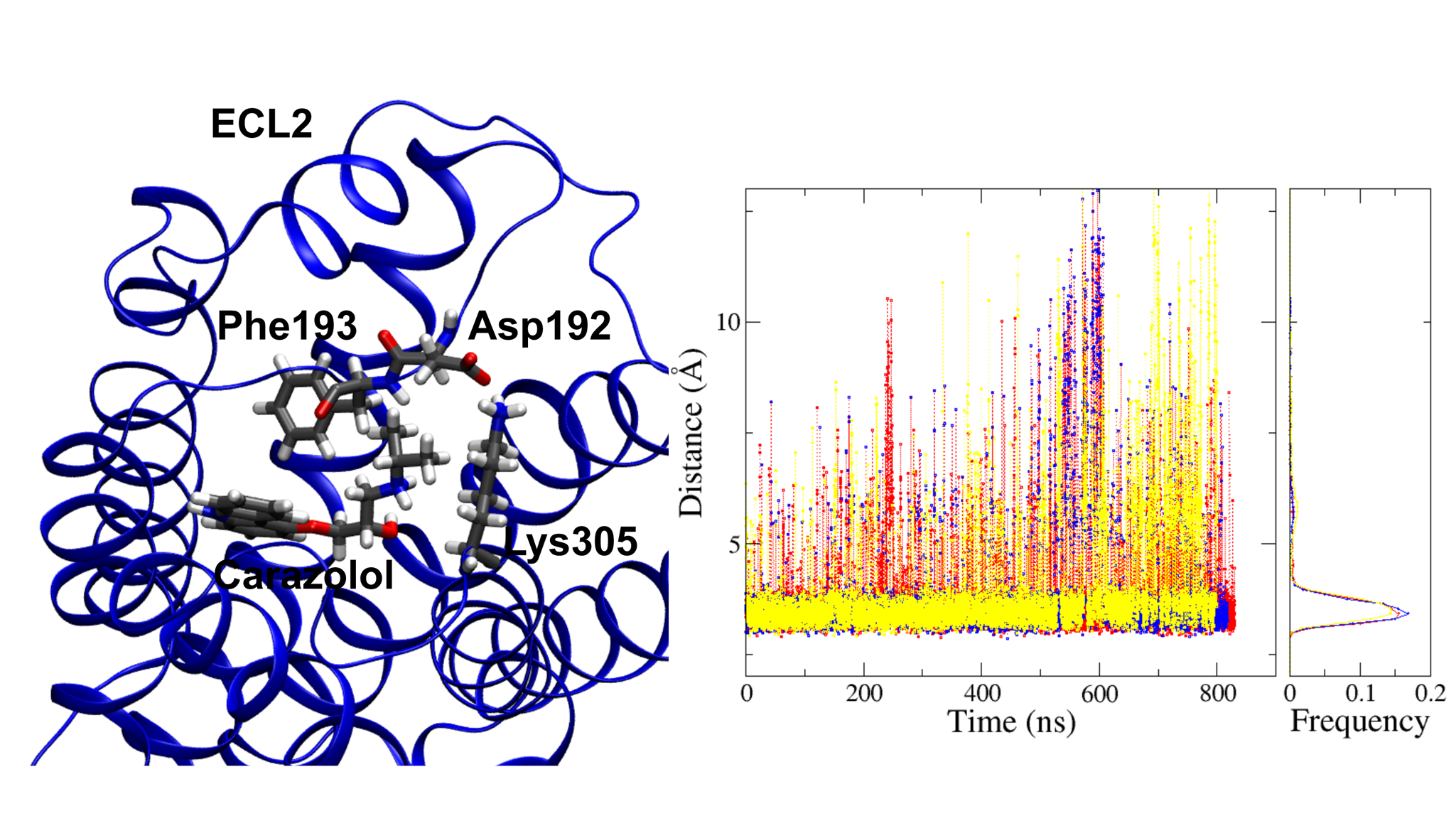

Supplement: Figure S3 — Left panel: Lys305-Asp192 salt bridge in β2AR. Time evolution of the Lys305-Asp192 salt bridge (Nζ@Lys305-Cγ@Asp192 distance) in MD simulations of carazolol-bound (blue), unliganded (yellow) and isoprenaline-bound (red) β2AR and respective frequency distribution. (red) β2AR. (1.33 MB TIF) [file pcbi.1001053.s006.tif]

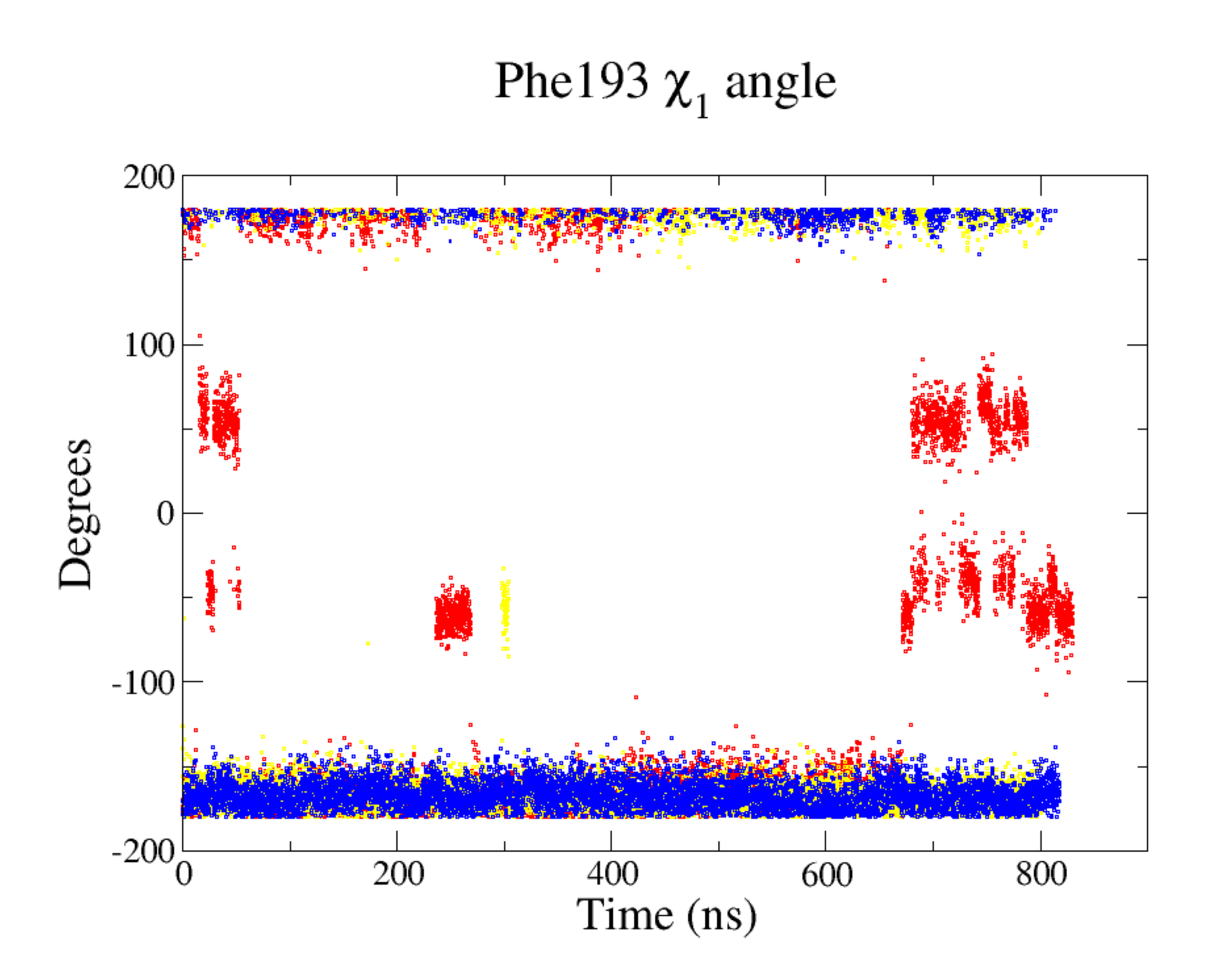

Supplement: Figure S4 — Time evolution of the Phe193 χ1 angle in MD simulations of carazolol-bound (blue), unliganded (yellow) and isoprenaline-bound (red) β2AR. (0.64 MB TIF) [file pcbi.1001053.s007.tif]

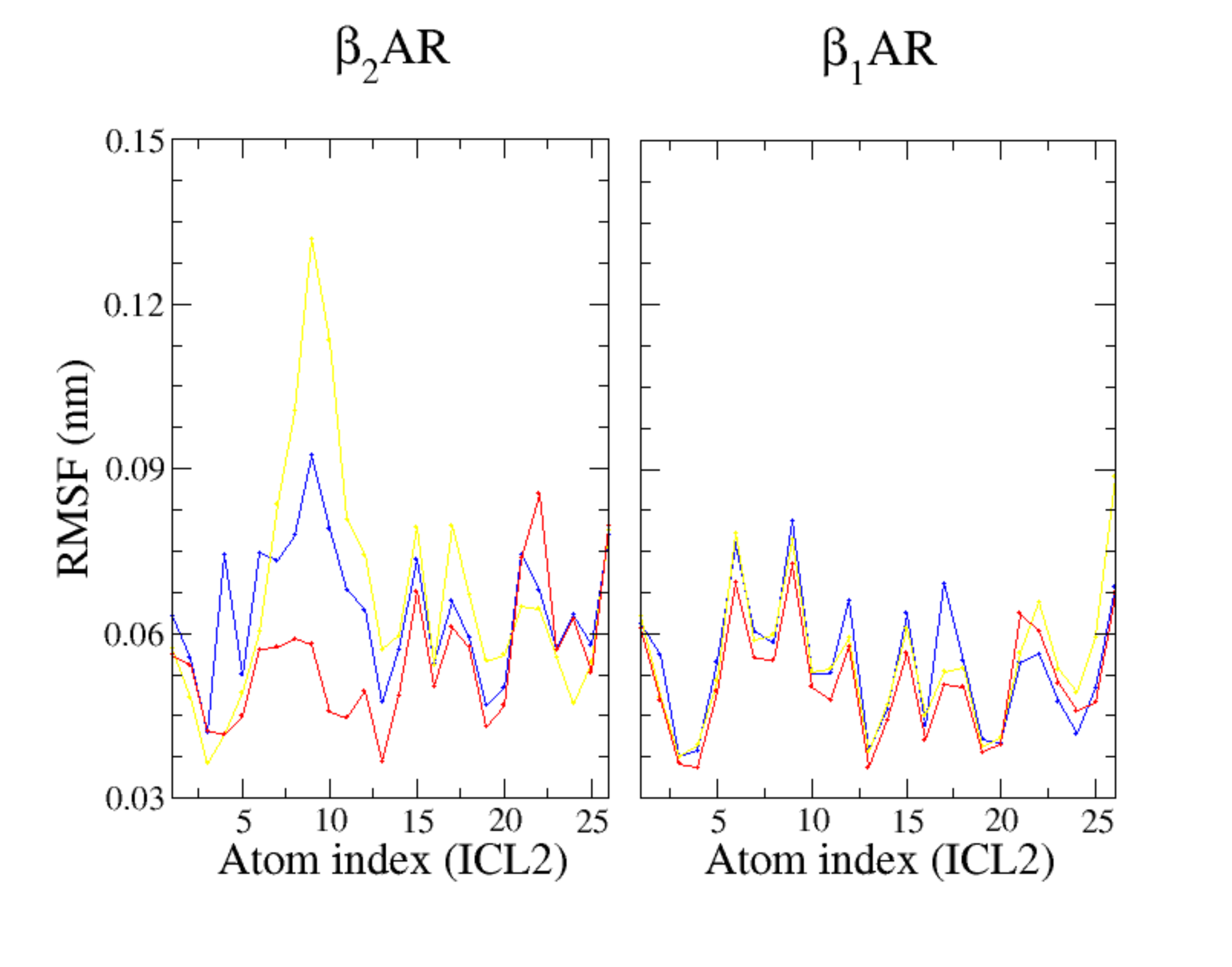

Supplement: Figure S5 — Root mean square fluctuations of intracellular loop 2 in MD simulations of antagonist-bound (blue), unliganded (yellow) and isoprenaline-bound (red) β2AR (left) and β1AR (right). Atom index #1 corresponds to His172 in β2AR and to His180 in β1AR. (0.49 MB TIF) [file pcbi.1001053.s008.tif]
