# Supplementary material for: Predicting Novel Binding Modes of Agonists to β Adrenergic Receptors Using All-Atom Molecular Dynamics Simulations
Source: PLoS Comput Biol. 2011 Jan 6;7(1):e1001053. doi: 10.1371/journal.pcbi.1001053 (PMC3017103; doi:10.1371/journal.pcbi.1001053)
Supplement: Table S1 — Hydrogen bond network between isoprenaline and adrenergic receptors. The table shows the percentage of N-O or O-O distances below 3.2 angstrom of the hydrogen bonds shown in Figures 3 and 4 after equilibration. (0.04 MB DOC) [file pcbi.1001053.s009.doc]

Supplementary information

Table S1. Hydrogen bond network between isoprenaline and adrenergic receptors. The table shows the percentage of N-O or O-O distances below 3.2 Å of the hydrogen bonds shown in Figures 3 and 4 after the equilibration period

| Receptor | Donor | Acceptor | Percentage of H-bond formation |
| --- | --- | --- | --- |
| β_2_AR | Tyr(7.35) | Asn(6.55) | 78% |
| β_2_AR | Asn(6.55) | Isoprenaline | 59% |
| β_2_AR | Asn(6.55) | Ser(5.43) | 75% |
| β_2_AR | Isoprenaline | Ser(5.42) | 97% |
| β_2_AR | Ser(5.47) | Ser(5.42) | 98% |
| β_2_AR | Ser(5.47) | Isoprenaline | 24% |
| β_1_AR | Asn(6.55) | Isoprenaline | 76% |
| β_1_AR | Asn(6.55) | Ser(5.43) | 56% |
| β_1_AR | Isoprenaline | Ser(5.42) | 69% |
| β_1_AR | Ser(5.47) | Ser(5.42) | 78% |
